# Disparities in the diagnosis and management of exocrine pancreatic insufficiency in resectable vs metastatic pancreatic cancer

**DOI:** 10.1093/oncolo/oyag084

**Published:** 2026-03-20

**Authors:** Peiyun Ni, Christian Baglini, Jessica Meurer, Lorraine Drapek, Sharvani Dhandibhotla, Yitong Liu, Fateh Bazerbachi, Colin Weekes, David T Ting, Avinash Kambadakone, Carlos Fernandez-del Castillo, Yasmin G Hernandez-Barco

**Affiliations:** Division of Gastroenterology, Massachusetts General Hospital, Boston, MA, 02114, United States; Massachusetts General Hospital Cancer Center, Boston, MA, 02114, United States; Massachusetts General Hospital Cancer Center, Boston, MA, 02114, United States; Massachusetts General Hospital Cancer Center, Boston, MA, 02114, United States; Division of Gastroenterology, Massachusetts General Hospital, Boston, MA, 02114, United States; School of Science, Engineering and Technology, Brandeis University, Waltham, MA, 02453, United States; CentraCare, Interventional Endoscopy Program, St. Cloud Hospital, Saint Cloud, MN, 56303, United States; Massachusetts General Hospital Cancer Center, Boston, MA, 02114, United States; Department of Medical Oncology, Massachusetts General Hospital, Boston, MA, 02114, United States; Massachusetts General Hospital Cancer Center, Boston, MA, 02114, United States; Department of Medical Oncology, Massachusetts General Hospital, Boston, MA, 02114, United States; Department of Radiology, Massachusetts General Hospital, Boston, MA, 02114, United States; Department of Surgery, Massachusetts General Hospital, Boston, MA, 02114, United States; Division of Gastroenterology, Massachusetts General Hospital, Boston, MA, 02114, United States

**Keywords:** exocrine pancreatic insufficiency, pancreatic enzymes, pancreatic neoplasms, nutritional support

## Abstract

**Background:**

Exocrine pancreatic insufficiency (EPI), a common complication of pancreatic cancer (PC), reduces quality of life and may shorten survival. While pancreatic enzyme replacement therapy (PERT) improves symptoms and outcomes, real-world patterns of EPI workup and PERT use across PC subtypes remain poorly described in the United States.

**Patients and Methods:**

We retrospectively analyzed 250 patients with resectable or metastatic PC from a single institution’s prospectively maintained registry (2013–2018), collecting data on clinical characteristics, EPI symptoms, fecal elastase testing, and PERT prescriptions. In addition to the retrospective analysis, a quality improvement intervention for EPI management was implemented (1/2021–1/2023), and outcomes were analyzed.

**Results:**

Among 250 patients, 97 underwent surgery for resectable disease and 153 received non-surgical management for metastatic PC. Exocrine pancreatic insufficiency symptoms occurred in 58% of metastatic and 68% of surgical patients. Fecal elastase testing was rarely performed (2% vs 9%, respectively). Pancreatic enzyme replacement therapy was prescribed to 46.5% of metastatic and 84% of surgical patients, but average doses were suboptimal (18 500 vs 20 000 USP units per meal; recommended: ≥40 000). Among those on PERT, symptom resolution was reported in 33% of metastatic and 44% of surgical patients. Contrasting with results from the retrospective analysis, the quality improvement intervention led to 90% of 41 participants being prescribed PERT at an average dose of 44 700 USP units per meal. Treated patients (74.1%) experienced complete resolution of EPI symptoms.

**Conclusion:**

Despite prevalent EPI symptoms in PC patients, fecal elastase testing was infrequently utilized, and PERT was often underdosed. Educational initiatives are needed to improve guideline adherence and optimize outcomes.

Implications for Practice Exocrine pancreatic insufficiency (EPI) is a common complication of pancreatic cancer (PC) that may impact quality of life and survival. This retrospective cohort study highlights gaps in current practice, including underuse of fecal elastase testing and suboptimal dosing of pancreatic enzyme replacement therapy. Examining both surgical and metastatic PC subgroups, it offers comprehensive insights into variation in EPI management across care trajectories. Given the findings, a quality improvement intervention was implemented and led to increased PERT prescribing and improved symptom resolution. Our study underscores the need for greater clinician awareness and adherence to guideline-based EPI management, while providing valuable workup and management recommendations to support clinicians in caring for PC patients with EPI.

## Introduction

Exocrine pancreatic insufficiency (EPI), defined as inadequate secretion of pancreatic enzymes required for digestion,^1^ is a common complication of pancreatic cancer (PC). These enzymes are essential for the breakdown of carbohydrates, fats, and proteins. In EPI, maldigestion and malabsorption may lead to diarrhea, steatorrhea, bloating, flatulence, and ultimately, malnutrition. In patients with PC, EPI contributes to weight loss, sarcopenia, vitamin deficiencies, poor tolerance of chemotherapy or surgery, and reduced survival.^2–5^

Multiple mechanisms contribute to EPI in PC patients, including parenchymal destruction by tumor, pancreatic ductal obstruction, surgical resection, and chemotherapy-induced injury.^6–8^ The likelihood of EPI varies based on tumor location (pancreas head vs body/tail), disease stage (local vs advanced), and treatment options (surgery vs chemotherapy).^9^ For example, pancreatic head tumor and surgical resection of the pancreatic head are associated with a high prevalence of EPI.^9^ Up to 50%-100% of patients with unresectable PC, and 40%-50% of patients with resectable disease preoperatively, may have EPI. After resection, this rises to over 75%.^10–12^

The fecal elastase test is a commonly used screening test for EPI. It measures the concentration of the specific isoform CELA3 (chymotrypsin-like elastase family) in a stool sample, which stays stable during intestinal transit and is readily detectable in stool. Compared to direct measurement of pancreatic secretion levels, it is simple to perform and serves as a useful, noninvasive test of pancreatic function.^13^^,^^14^ However, the test has low sensitivity for mild EPI,^15^ and requires a solid or semisolid stool specimen,^16^ as watery stool can dilute the sample and lead to false-positive results. Therefore, in patients with a high pretest probability of EPI, empiric treatment should be started with ongoing symptom monitoring, regardless of test results.

Pancreatic enzyme replacement therapy (PERT) with pancrelipase is the key treatment of EPI. It improves fat and protein digestion, alleviates symptoms, and may prevent malnutrition in post-resection patients.^17^ Pancreatic enzyme replacement therapy is well tolerated, easy to administer, and has been associated with improvements in both quality of life and overall survival.^18–23^

Despite its clinical benefits, PERT is underutilized in PC patients with EPI. In a study based on a national database in the United States from 2001 to 2013, only 1.9% PC patients had testing for EPI and 21.9% had a prescription for PERT.^4^ A more recent retrospective analysis in 2023 found that only 27.3% patients with PC or chronic pancreatitis and documented EPI received PERT.^24^ In patients on PERT, the American Gastroenterological Association (AGA) guidelines recommend that at least 40 000 USP units of lipase should be given with each meal and one-half of that with snacks.^16^ Adequate dosing and correct administration are essential, as symptom resolution strongly correlates with proper use.^25^ While European studies have highlighted considerable variability in PERT prescribing practices and frequent underdosing,^26^^,^^27^ data from the United States, particularly comparing different PC subtypes, remain limited.

To address this gap, we aimed to describe EPI workup and PERT prescribing patterns in a real-world cohort of PC patients at a US tertiary medical center. Our goal was to evaluate current clinical practices and highlight areas for potential quality improvement in EPI management.

## Methods

### Study design

We performed retrospective analysis after identifying PC patients from a prospectively maintained registry between 2013 and 2018 at Massachusetts General Hospital (MGH), a tertiary care center in the United States. The database contained basic characteristics of patients who had PC (age at diagnosis, sex, and race), disease stage (metastatic disease vs surgically managed), oncologic treatment (chemotherapy and surgery status), symptoms, diagnostic testing results, and prescribed medications.

**Figure 1.**
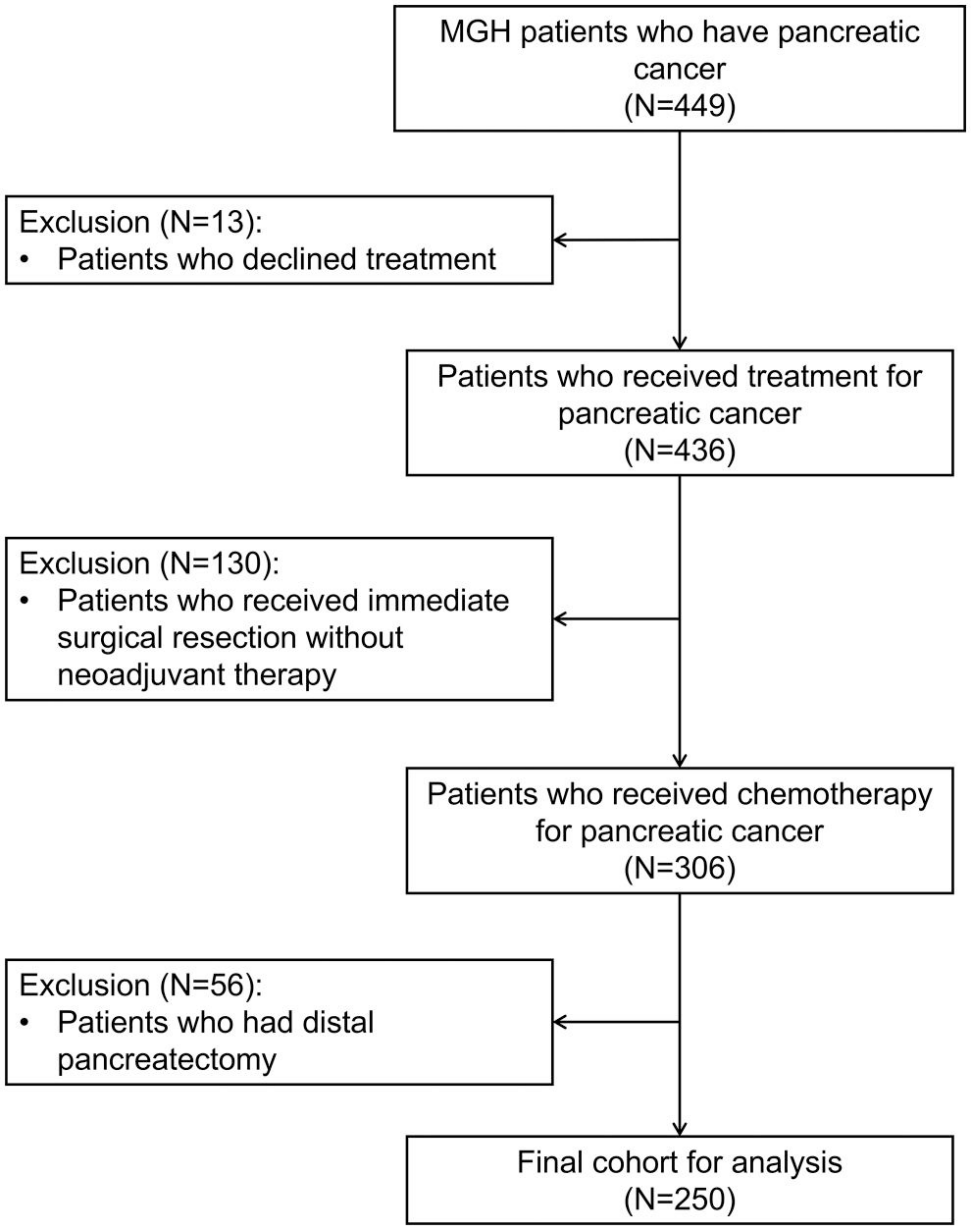
Patient selection criteria using the database from Massachusetts General Hospital.

A total of 449 patients with PC were initially identified. We excluded patients who declined cancer treatment (*n* = 13), those who underwent immediate surgical resection without neoadjuvant therapy (*n* = 130), and those who underwent distal pancreatectomy (*n* = 56), given the lower associated risk of EPI with this surgical procedure. The final study cohort included 250 patients. Inclusion and exclusion criteria were summarized in Figure 1. Informed consent for data inclusion was obtained via in-person physician discussion, and the study protocol was approved by the institutional review board at MGH.

### Exocrine pancreatic insufficiency workup documentation

Exocrine pancreatic insufficiency symptoms were recorded through chart review, only if they were explicitly mentioned in medical notes. These include weight loss, diarrhea, and steatorrhea. Resolution of symptoms with PERT was also documented if indicated in medical notes. As the fecal elastase test is commonly used as the initial screening test for EPI, its utilization and corresponding results were documented for each patient. Per AGA guidelines, confirmed EPI was defined as a fecal elastase level <100 μg/g of stool in the presence of clinical symptoms.^16^

### Pancreatic enzyme replacement therapy prescription and related information

Pancreatic enzyme replacement therapy prescription data were collected for all patients by chart review. Data collected included the dispensation date, brand (Creon, Zenpep, Pancreaze), capsule strength (USP units of lipase), and number of capsules prescribed. Patients were classified as receiving PERT if it was prescribed at any point during their clinical care. Compliance data were obtained from clinical notes when clinicians or nutritionists explicitly documented patient adherence to PERT. When such documentation was unavailable, the medication dispense history was used. Because pancrelipase is designed to release enzymes at a pH of approximately 5.5 or above,^28^ pH-altering medications and comorbid conditions may also affect the efficacy of PERT. Therefore, we collected data on concurrent prescription of proton pump inhibitors (PPIs), histamine-2 (H2) receptor antagonists, lactic acid bacterial preparations, and digestive enzyme supplements, as well as a history of peptic ulcer disease based on endoscopy reports, among patients who were prescribed PERT.

### Quality improvement intervention

In addition to the retrospective analysis, we also implemented a quality improvement intervention from January 2021 through January 2023, beginning with a nurse practitioner (NP)-initiated assessment and plan at PC patient entry into the ambulatory oncology setting. This included EPI diagnosis, nutrition referral, and PERT prescribing. Many patients received empiric PERT without fecal elastase testing, due to a high pretest probability of EPI or severe watery diarrhea that hindered solid stool collection required for the test. Virtual or in-person NP follow-up visits were completed every 2 months throughout the entirety of the treatment period per standard of care. All patients received nutrition consultation with a registered dietitian specializing in oncology. The initial consultation visit involved a comprehensive nutrition assessment and an in-depth discussion of the patient’s condition, addressing factors related to chemotherapy, underlying disease, comorbidities, symptom optimization, and EPI. In addition, each patient received follow-up nutrition consultation per the recommendation with a registered dietitian to ensure ongoing support, counseling, resources, and nutritional management throughout the patient’s disease trajectory. Exocrine pancreatic insufficiency-related symptoms of patients were documented before and after the intervention.

### Statistical analysis

Descriptive statistics were performed to assess the baseline characteristics, prevalence of EPI symptoms, frequency of fecal elastase test, and PERT use patterns. Categorical variables were reported as proportions (%) and analyzed using Fisher’s exact test. Continuous variables were presented as mean with standard deviation (SD), and analyzed using the Wilcoxon rank sum test. A *P*-value of <0.05 was considered statistically significant. Statistical analyses were performed using R version 4.4.1 (R Foundation for Statistical Computing, Vienna, Austria; http://www.R-project.org/).

## Results

### Baseline characteristics

A total of 250 patients met the inclusion criteria. Of these, 153 received non-surgical management for metastatic PC, and 97 underwent surgical resection for locally advanced PC. Among all patients, 51% were female, and 90.4% were white. The mean age at the time of PC diagnosis for all patients was 67.4 years old. The patient characteristics were summarized in Table 1.

**Table 1.** Baseline characteristics, EPI symptoms, fecal elastase testing and PERT use of patients.

Variables	Metastatic cancer (*N* = 153)	Surgically managed (*N* = 97)	*P* **-value**
**Sex**			0.70
**Female**	80 (52%)	48 (49.5%)	
**Male**	73 (48%)	49 (50.5%)	
**Race**			0.88
**White**	138 (90.2%)	88 (90.7%)	
**Black**	6 (3.9%)	2 (2.1%)	
**Asian**	3 (2%)	2 (2.1%)	
**Unknown**	6 (3.9%)	5 (5.2%)	
**Average age at the time of PC diagnosis (SD)**	68.8 (11.5)	66 (8.4)	0.07
**EPI symptoms**			
**Weight loss**	88 (58%)	66 (68%)	0.11
**Diarrhea**	83 (54%)	52 (54%)	0.99
**Steatorrhea**	18 (12%)	20 (21%)	0.07
**Fecal elastase test performed**	3 (2%)	9 (9%)	0.01
**PERT prescription**	71 (46.5%)	82 (84%)	<0.001
**PERT formulation**			0.62
**Creon**	68 (44.4%)	67 (70%)	
**Zenpep**	3 (2%)	1 (1%)	
**Pancreaze**	0 (0%)	1 (1%)	
**Average PERT dose (SD)**	18 500 units per meal (7790)	20 000 units per meal (8109)	0.25
**Concurrent medication use with PERT**			
**Proton pump inhibitor**	51 (71.8%)	67 (81.7%)	0.18
**Histamine-2 receptor antagonist**	4 (5.6%)	8 (9.8%)	0.38
**Lactic acid bacterial supplementation**	4 (5.6%)	12 (14.6%)	0.11
**Other digestive enzymes**	1 (1.4%)	2 (2.4%)	0.99
**History of peptic ulcer disease among patients on PERT**	2 (2.8%)	7 (8.5%)	0.18
**Resolution of symptoms with PERT**	50 (33%)	43 (44%)	0.03

Abbreviations: EPI, exocrine pancreatic insufficiency; PERT, pancreatic enzyme replacement therapy; PC, pancreatic cancer; SD, standard deviation.

### Exocrine pancreatic insufficiency symptoms and diagnostic workup

At least one EPI-related symptom was documented in 58% (*n* = 89) of patients with metastatic PC and 68% (*n* = 66) of those who underwent surgery. Diarrhea was the most common symptom, occurring in 54% of patients in both groups. Weight loss was more prevalent in the surgical group but did not reach statistical significance (68% vs 58%, *P* = 0.11), as was steatorrhea (21% vs 12%, *P* = 0.07) (Table 1).

Fecal elastase testing was rarely utilized. Only 2% (*n* = 3) of patients with metastatic PC and 9% (*n* = 9) of surgical patients with locally advanced PC had fecal elastase testing (Table 1).

### Pancreatic enzyme replacement therapy prescription patterns

Pancreatic enzyme replacement therapy was prescribed for 46.5% (*n* = 71) of metastatic PC patients and 84% (*n* = 82) of surgical patients (*P* < 0.001). Most prescriptions were given empirically, without fecal elastase confirmation. Creon was the most commonly prescribed formulation, used in 44.4% (*n* = 68) of metastatic and 70% (*n* = 67) of surgical patients. Fewer patients received Zenpep or Pancreaze. The average prescribed dose was 18 500 USP units of lipase per meal in the metastatic group and 20 000 USP units per meal in the surgical group (Table 1)—both lower than the AGA-recommended 40 000 USP units per meal. The compliance rates were 36.6% (*n* = 26) in the metastatic group and 62.2% (*n* = 51) in the surgical group. However, these results were influenced by the lack of compliance data in electronic records (57.5% missing in the metastatic group and 25.6% missing in the surgical group).

Among patients on PERT, PPIs were commonly used, with 71.8% of patients in the metastatic group and 81.7% in the surgical group receiving them. Proton pump inhibitors may enhance PERT efficacy by suppressing gastric acid, as an alkaline pH promotes enzyme release.^28^ Fewer patients were on H2 receptor antagonists (5.6% in the metastatic group and 9.8% in the surgical group) or lactic acid bacterial supplementation (5.6% and 14.6%, respectively). Only 2 patients in total were prescribed other digestive enzymes, and both were on lactase. Two patients (2.8%) in the metastatic group and 7 patients (8.5%) in the surgical group had peptic ulcer disease.

### Symptom change with PERT

Among patients who were prescribed PERT, complete resolution of EPI symptoms was documented in 33% (*n* = 50) of metastatic cases and 44% (*n* = 43) of surgical cases (Table 1). Of the 153 patients with PERT prescriptions, 4 (2.6%) had no recorded EPI symptoms, and 17 (11.1%) had no documentation of treatment effect, suggesting limited missing data.

### Outcome of quality improvement intervention

**Figure 2.**
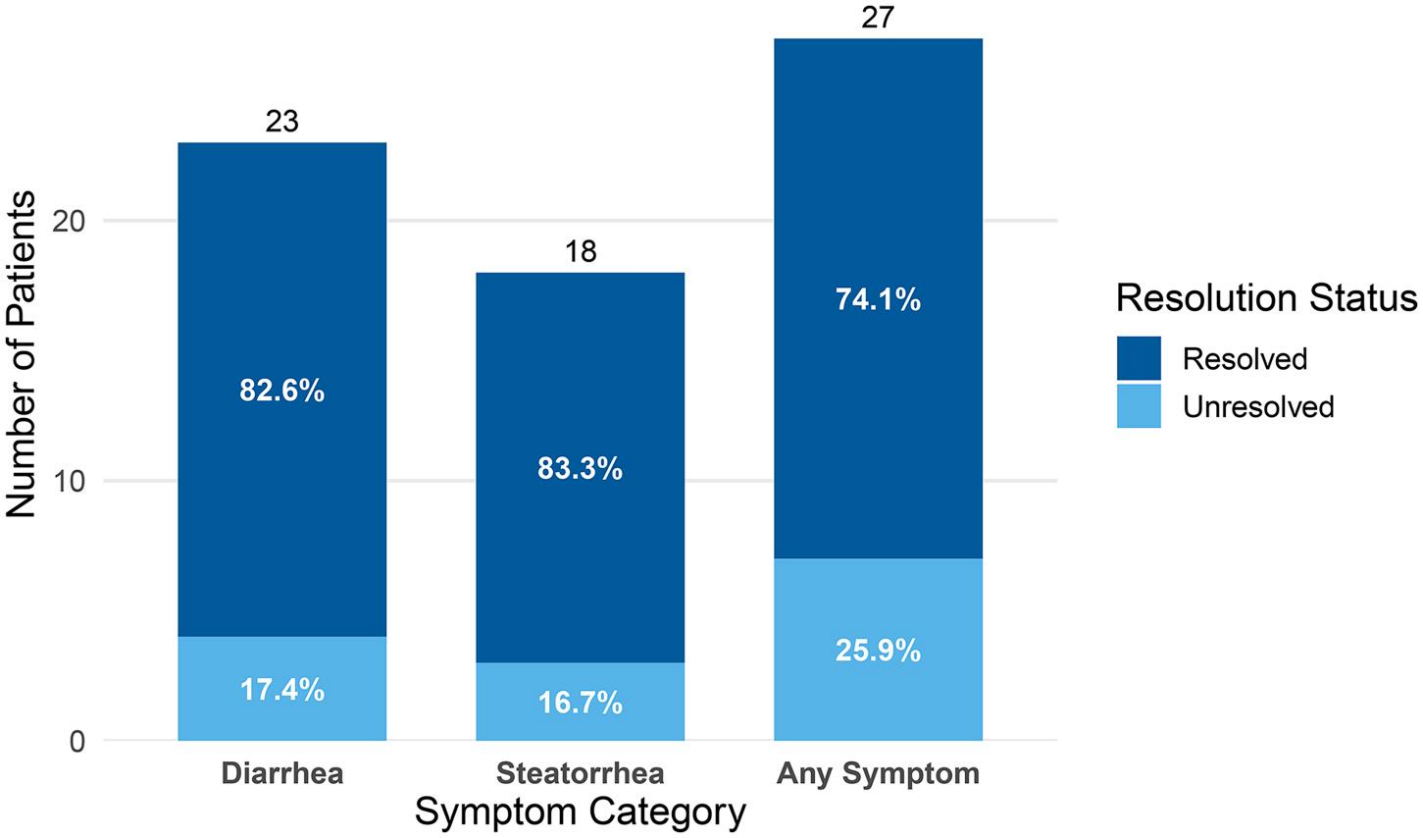
Exocrine pancreatic insufficiency symptom resolution status after PERT in the intervention group.

A total of 41 PC patients were included in the quality improvement intervention. All patients completed the nutrition consultation. Pancreatic enzyme replacement therapy was prescribed for 90% of patients, and within the first 3 months of PC diagnosis for 65% of patients. The average initial PERT dose prescribed to patients was 44 700 USP units per meal. While many patients received empiric PERT without fecal elastase testing, due to high pretest probability of EPI or inability to provide a solid stool sample from severe diarrhea, 3 patients were tested, and all had fecal elastase levels <15 μg/g, consistent with EPI. Among 40 patients with available documentation on symptoms before and after PERT, 57.5% (*n* = 23) had diarrhea, and 45% (*n* = 18) had steatorrhea before PERT. Among patients with diarrhea, 82.6% (*n* = 19) experienced symptom resolution after PERT treatment, compared with 17.4% (*n* = 4) who did not (*P* = 0.002). Similarly, 83.3% of patients with steatorrhea (*n* = 15) had symptom resolution, whereas 16.7% (*n* = 3) did not (*P* = 0.005). In total, 74.1% of treated patients with any EPI symptom (diarrhea, steatorrhea, or both) experienced complete symptom resolution, which was 50% of all patients (*n* = 20), compared with 25.9% (*n* = 7) who did not (*P* = 0.01) (Figure 2).

## Discussion

Exocrine pancreatic insufficiency is a common but often under-recognized complication in patients with PC. It contributes significantly to malnutrition, weight loss, and reduced treatment tolerance, factors that can directly affect prognosis.^2–5^ The likelihood of EPI development varies based on tumor subtype, disease stage, and therapeutic interventions. While PERT alleviates EPI symptoms and may confer survival benefits,^20–23^ its clinical application in PC patients remains suboptimal.

Our study showed that EPI symptoms were common, which occurred in more than half of the metastatic PC patients and patients who underwent surgery. Fecal elastase stool study, the screening test for EPI, was performed in less than 10% of patients in both groups. This was consistent with existing studies demonstrating that only a small number of PC patients received testing for EPI.^4^^,^^29^ Higher prescription rates of PERT may partly reflect guideline-recommended empiric use in patients with a high pretest probability of EPI, bypassing confirmatory testing. However, testing should be encouraged when clinical findings conflict with the initial suspicion to establish diagnostic certainty. As a general rule, in PC patients with lower pretest probability of EPI—for example, those with small, focal tumors in the distal pancreas, fecal elastase test can effectively rule out EPI with a false negative rate as low as 1.1%. In patients with high pretest probability of EPI, the false negative rate increases to 10%, so empiric PERT should be started even if the fecal elastase level is normal.^13^ If symptoms persist after the initiation of PERT, the dose should be increased and administration reviewed to ensure it is taken with meals. Persistent symptoms despite high-dose PERT warrant a trial of PPIs. If there is still no improvement, alternative causes should be evaluated, such as chemotherapy-induced diarrhea or small intestinal bacterial overgrowth (SIBO).^30^

We also found that PERT was prescribed more frequently to patients who underwent surgical resection (84%) compared to those with metastatic disease (46.5%) (*P* < 0.001). This likely reflects a higher clinical suspicion of EPI following pancreaticoduodenectomy, where exocrine dysfunction is nearly universal.^29^ Although patients with metastatic PC were more often managed by medical oncologists, it remains unclear whether the higher PERT prescription rate among surgical patients reflects a greater tendency among surgeons to prescribe PERT, or if it is primarily driven by the high risk of EPI following pancreatic head resections.

Creon was the most frequently prescribed pancrelipase formulation at our institution, compared to alternatives such as Zenpep or Pancreaze. Although the exact reason for this cannot be confirmed based on available data, a possible explanation is that Creon appears first on the formulary in our electronic medical record (EMR) system when physicians order pancrelipase, and the EMR provides a dosing recommendation for Creon. These features may have contributed to the higher utilization of Creon at our institution, underscoring how formulary design and embedded clinical decision support tools may influence prescribing patterns.

Importantly, we found that PERT was underdosed in both groups. The average prescribed doses (18 500–20 000 USP units per meal) were consistently below the AGA-recommended 40 000 USP units per meal.^4^ Suboptimal dosing may explain the modest rates of symptom resolution reported (33% in metastatic, 44% in surgical patients), and reinforces the importance of both initiating and appropriately titrating PERT. Prior studies have documented similar issues with underdosing and poor adherence to guideline-based PERT regimens.^4^^,^^26^^,^^31^ Physician undertreatment is likely multifactorial, driven by limited education, concerns about overdosing, and challenges in accurately diagnosing EPI. First, education regarding the benefits of PERT should be emphasized. A US survey of pancreatic surgeons on their use of PERT for EPI in PC showed that the prescription patterns were strongly influenced by surgeons’ perceptions of PERT’s impact on survival. Surgeons who routinely prescribed PERT had greater confidence in its ability to improve survival outcomes than under-prescribers.^29^ Second, concerns about dosing may lead to underutilization. Creon is available in multiple dose formulations, and unless clinicians review dosing recommendations, they may feel more comfortable prescribing a lower dose. This tendency contributes to underdosing, which is also commonly observed in other EPI-related conditions, such as chronic pancreatitis.^4^^,^^31^ Third, diagnosing EPI in patients with PC can be challenging, because diarrhea is often multifactorial and potentially related to SIBO, chemotherapy, postoperative dumping, or other causes. Diagnostic uncertainty may discourage clinicians from prescribing PERT or using guideline-recommended doses. Given the prevalence of EPI and the minimal risk of guideline-based therapy, our findings reveal a gap in provider education and clinical practice required for timely diagnosis and appropriate treatment of EPI, pointing to the need for improved EPI management.

Building on this identified gap, we implemented a quality improvement initiative that addressed EPI diagnosis, nutrition referral, and appropriate EPI prescribing for PC patients. Despite similar rates of diarrhea and even higher rates of steatorrhea than the patient population in our retrospective analysis, the cohort experienced a higher rate of complete EPI symptom resolution. Our results demonstrate that better recognition, timely diagnosis, and adequate treatment of EPI can lead to better patient outcomes. In addition, a registered dietitian should be included in the multidisciplinary care team to improve EPI management. Dietitians play a crucial role in monitoring patients’ nutritional status and identifying deficits early. While oncologists, surgeons, and gastroenterologists remain essential for diagnosing and managing EPI, having a dedicated dietitian ensures timely evaluation of nutritional needs and interventions. This includes assisting with PERT counseling and providing tailored dietary recommendations, allowing other physicians to focus on their primary areas of care without EPI being overlooked. Such an approach may improve EPI detection, enhance appropriate PERT administration, and ultimately lead to better outcomes.

This study has several strengths. It adds to the limited literature characterizing real-world PERT use among PC patients in the United States. The inclusion of both surgical and metastatic subgroups provides insight into variable practice patterns across treatment paradigms. Moreover, we implemented a quality improvement intervention to optimize EPI diagnosis and treatment for PC patients at a tertiary medical center, demonstrating that improved PERT utilization is associated with improved EPI symptom resolution. Our study has several limitations that should be acknowledged. While the database was prospectively maintained, the analysis was retrospective and reliant on documentation in clinical notes, which may bias the documentation of symptoms or therapy response. Compliance data were also sparse, limiting the ability to assess adherence. Additionally, the study was based on records from only one tertiary medical center, limiting its generalizability. We also did not evaluate PERT administration timing or duration—factors that could influence outcomes. Finally, the specialty of the prescribing provider (eg, surgeon vs oncologist vs gastroenterologist) was not consistently recorded but may play a role in PERT utilization.

Future work should explore whether prescribing patterns differ by provider specialty and whether targeted educational initiatives improve dosing practices and symptom outcomes. Prospective studies incorporating symptom tracking and nutritional markers would also be valuable.

## Conclusion

Exocrine pancreatic insufficiency is common in both surgically treated and metastatic PC. While PERT offers symptom relief and potential survival benefits, this study found wide variation in its use and dosing across patient groups. The higher prescription rate in surgical patients may reflect the expected risk of EPI after pancreatic head resection, but under-recognition in the metastatic setting remains a concern. Standardized clinical pathways and educational efforts are needed to ensure consistent, guideline-based management of EPI in PC.

## Data Availability

The data used to support the findings of this study are available after deidentification from the corresponding author upon reasonable request.
